# Recombinant mussel protein Pvfp-5β: A potential tissue bioadhesive

**DOI:** 10.1074/jbc.RA119.009531

**Published:** 2019-07-10

**Authors:** Radha Santonocito, Francesca Venturella, Fabrizio Dal Piaz, Maria Agnese Morando, Alessia Provenzano, Estella Rao, Maria Assunta Costa, Donatella Bulone, Pier Luigi San Biagio, Daniela Giacomazza, Alessandro Sicorello, Caterina Alfano, Rosa Passantino, Annalisa Pastore

**Affiliations:** ‡ Istituto di Biofisica, Consiglio Nazionale delle Ricerche, Palermo I90146, Italy; § University of Palermo, Palermo I90128, Italy; ‖ University of Salerno, Salerno I84084, Italy; ¶ Fondazione Ri.MED, Palermo I90133, Italy; ** King's College London, London SE59RT, United Kingdom; ‡‡ UK Dementia Research Institute at King's College London, London SE59RT, United Kingdom

**Keywords:** adhesion, biomaterials, biophysics, epidermal growth factor (EGF), structural biology, adhesion proteins, EGF-like motifs, Marine proteins

## Abstract

During their lifecycle, many marine organisms rely on natural adhesives to attach to wet surfaces for movement and self-defense in aqueous tidal environments. Adhesive proteins from mussels are biocompatible and elicit only minimal immune responses in humans. Therefore these proteins have received increased attention for their potential applications in medicine, biomaterials, and biotechnology. The Asian green mussel *Perna viridis* secretes several byssal plaque proteins, molecules that help anchoring the mussel to surfaces. Among these proteins, protein-5β (Pvfp-5β) initiates interactions with the substrate, displacing interfacial water molecules before binding to the surface. Here, we established the first recombinant expression in *Escherichia coli* of Pvfp-5β. We characterized recombinant Pvfp-5β, finding that despite displaying a CD spectrum consistent with features of a random coil, the protein is correctly folded as indicated by MS and NMR analyses. Pvfp-5β folds as a β-sheet–rich protein as expected for an epidermal growth factor-like module. We examined the effects of Pvfp-5β on cell viability and adhesion capacity in NIH-3T3 and HeLa cell lines, revealing that Pvfp-5β has no cytotoxic effects at the protein concentrations used and provides good cell-adhesion strength on both glass and plastic plates. Our findings suggest that the adhesive properties of recombinant Pvfp-5β make it an efficient surface-coating material, potentially suitable for biomedical applications including regeneration of damaged tissues.

## Introduction

It was proposed in the 1990s that animals such as mussels, sandcastle worms, and geckos are great potential sources of nontoxic adhesive biomaterials that have the additional advantage to be suited for wet environments (1, 2). These strong and water-insoluble adhesion properties have attracted increasing interest for potential applications in regenerative medicine, biotechnology, and material science (3–6). In particular, mussels have received significant attention especially because of their ability to adhere so tightly to their substrates to resist also turbulent tidal conditions (7–9). Mussel adhesion is possible through the secretion of a protein-based holdfast (byssus), which has evolved to anchor the mussel shell to underwater rocks and other substrates (10–12). The byssus consists of a bundle of threads intertwined together and forming a filament (13, 14). The end of each thread forms an adhesive plaque that contains a water-resistant glue and allows anchoring to entirely different substrates including glass, Teflon, metal, and plastic (15, 16). Chemically, the byssus is composed of mussel-adhesion proteins that are synthesized in the mussel foot. Six mussel foot proteins (mfps)^4^ have been identified in the *Mytilus* genus (mfp-2, -3S, -3F, -4, -5, and -6) (17). A particularly attractive property of these proteins is their inherent biodegradable nature, which makes them environmentally friendly (18). They are also good candidates as medical adhesives because they are usually nontoxic to the human body and do not easily elicit strong immune response (19–22). Mfps are thought to acquire their adhesive properties through a post-translational modification that consists in the enzymatic hydroxylation of tyrosine to 3,4-dihydroxyphenyl-l-alanine (DOPA) (23). In a recent study the abundance and proximity of catecholic DOPA and lysine residues suggest a synergistic interplay in adhesion (24). DOPA residues enable cross-linking of the proteins by oxidative conversion to *o*-quinone resulting in the filamentous byssus (25–27). Although preliminary studies have suggested that mussel-adhesion protein analogues without DOPA have reduced ability for adhesion, firm confirmation of these result is still lacking because of the insufficient characterization of the proteins, especially in their non-DOPA bound form because of difficulties in producing the proteins in their recombinant form.

Three mfps were recently identified in the Asian green mussel *Perna viridis* foot (28–30) by RNA-Seq integrated with proteomic analysis (31), Pvfp-3, Pvfp-5β, and Pvfp-6. They have molecular masses ranging between 5.3 and 11.2 kDa. Saline-induced adhesive secretions from mussel foot of *P. viridis* showed that, among them, the Pvfp-5β variant in the 8–10 kDa range is secreted first, typically within 10 s after saline injection. It was hypothesized that this protein is also the first protein to initiate interaction with the substrate making it a system of particular interest (32). Sequence analysis identified two tandem EGF motifs along the Pvfp-5β amino acid sequence that shares 47–50% identity with the EGF repeats of the Notch ligand Δ-like 1 protein (33). Such elevated identity makes it convincing to expect a tight similarity of the fold. EGF motifs are all-β-proteins that are characterized by three conserved disulfide bridges. The presence of EGF-like modules in mfps is not unique of Pvfp-5β because other mussel-adhesive proteins of various species, such as Mgfp-2 of *Mytilus galloprovincialis*, have several copies of EGF-like motifs (34, 35).

In this study, we describe the first implementation of the successful production of recombinant Pvfp-5β and its characterization by circular dichroism (CD), nuclear magnetic resonance (NMR), and dynamic light scattering (DLS). We show that the protein, which was produced as inclusion bodies, can be refolded in a species with the disulfide bridges expected on the basis of sequence homology correctly formed. We also prove for the first time that, despite having features that are not typical of an all-β-protein, Pvfp-5β folds as a β-rich protein as expected for an EGF-like module. We analyzed in detail the aggregation and adhesion properties of Pvfp-5β as part of a longer-term involvement aimed at the characterization of mfp-based biomaterial. Our results show that Pvfp-5β has intrinsic adhesive properties also in the absence of DOPA attachment. These properties make the protein an excellent and efficient surface-coating material that could be used for biomedical applications including the regeneration of damaged tissues.

## Results

### Finding the conditions for reproducible production of recombinant HT-Pvfp-5β

Several attempts were done to optimize the production of recombinant Pvfp-5β as this is the prerequisite of any future study on this protein. Recombinant Pvfp-5β was first expressed as a thioredoxin fusion protein with a tobacco etch virus (TEV) cleavable site just before the Pvfp-5β (phTt-Pvfp-5β). The protein was expressed in inclusion bodies from which it was solubilized under denaturing and reducing conditions in 8 m urea and refolded by slowly removing the denaturing agent dialyzing in phosphate buffer at pH 7.4. This did not result in a correctly folded sample as judged by MS analysis (data not shown). The construct was then modified and subcloned as a TEV protease cleavable N-terminal His-tag protein (HT-Pvfp-5β), which, although still resulting in inclusion bodies, would allow us to exclude unwanted tags. After solubilizing the inclusions bodies, purification was carried out under denaturing conditions with a single step affinity purification by immobilized-metal affinity chromatography because HT-Pvfp-5β was already relatively pure in the washed inclusion bodies (Fig. S1, *A* and *B*). To allow correct refolding of the protein, urea was gradually removed by sequential dialysis in the presence of both reduced and oxidized GSH. A final dialysis was performed in 5% acetic acid. An acidic pH mimics the conditions observed in the distal depression cavitation during mussel adhesion (17). His-tag removal by TEV protease digestion was achieved in phosphate buffer at pH 7.4 or sodium acetate buffer at pH 5.6 (Fig. S1*C*, *lanes 2* and *3*). All the experiments described hereafter were repeated with and without the tag. We did not observe noticeable differences (data not shown). We decided to show the data obtained with the tagged protein for consistency and historical reasons. The apparent molecular mass of HT-Pvfp-5β on SDS-PAGE was 13 kDa *versus* the predicted molecular mass of 9.4 kDa. The difference is likely the consequence of the basic pI value (9.2) and a possible nonglobular shape of the protein.

### Identification of the disulfide patterns by MS

**Figure 1.**
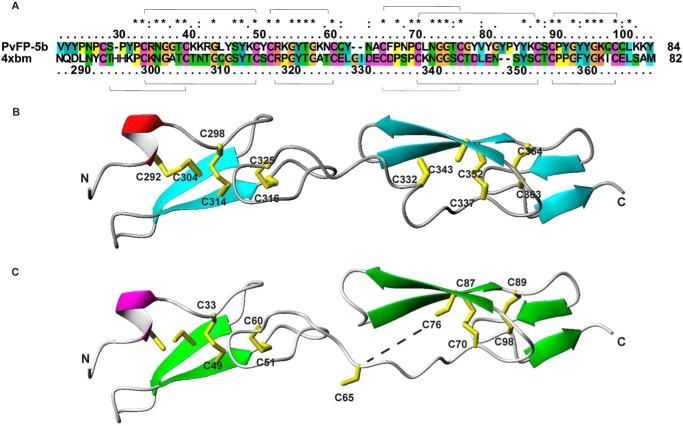
**Structural prediction of the fold of HT-Pvfp-5β based on sequence similarity.** *A,* sequence alignment between Pvfp-5β (*top*) and the Notch ligand Δ-like 1 protein (*bottom*). The sulfur bridges observed in Pvfp-5β are reported as *lines on the top* of the alignment, whereas the bridges observed in Notch ligand Δ-like 1 are reported on the *bottom*. All 12 cysteines are conserved. *B,* the crystal structure of Notch ligand Δ-like 1 protein (4xbm). The sulfur bridges observed in the Notch structure are indicated. *C,* comparative model of Pvfp-5β using 4xbm as a structural template. The sulfur bridges observed experimentally by MS are indicated either explicitly or with a discontinuous line for the pair Cys^65^–Cys^76^ that in the model is too far to be bridged.

A MS-based approach on the final product was used to characterize the disulfide bond pattern of the protein. ESI-MS analysis of carboxyamidomethylated HT-Pvfp-5β indicated that only two of the 12 cysteines were not involved in disulfide bridges (measured molecular weight 12021.1, theoretical molecular weight of tetra-carboxyamidomethylated HT-Pvfp-5β carrying 5 disulfide bridges 12020.6). Enzymatic digestion of the modified protein and the subsequent LC-MS/MS analysis of the resulting peptides allowed description of the coupling of the other 10 cysteines (Table 1 and Fig. 1*A*): identification of the disulfide-bridged peptides (32–34)–(49–50) indicated the presence of a covalent bond involving Cys^33^ and Cys^49^. Similarly, the peptides (51–52)–(59–62), (63–66)–(72–78), (67–71)–(87–88), and (89–91)–(97–100) were detected, demonstrating the presence of the disulfide bridges Cys^51^–Cys^60^, Cys^65^–Cys^76^, Cys^70^–Cys^87^, and Cys^89^–Cys^98^, respectively. Cys^28^ and Cys^39^ were not involved in a disulfide bridge and were found as carboxyamidomethylated peptides-(24–31) and (35–40), respectively.

**Table 1:** **Mass spectrometry-based identification of cysteine-containing peptides generated by trypsin and chymotrypsin digestion of carboxyamidomethylated HT-Pvfp-5β**

Peptide	Experimental molecular weight	Theoretical molecular weight	Involved cysteines
24–31	996,409	996,405	Cys^28^–CAM*^a^*
(32–43)–(49–50)	656,281	656,255	Cys^33^–Cys^49^
35–40	635,282	635,270	Cys^39^–CAM*^a^*
(51–52)–(59–62)	801,333	801,305	Cys^51^–Cys^60^
(63–66)–(72–78)	1121,469	1121,404	Cys^65^–Cys^76^
(67–71)–(87–88)	568,326	568,303	Cys^70^–Cys^87^
(89–91)–(97–100)	869,374	869,356	Cys^89^–Cys^98^

*^a^* CAM, carboxyamidomethylated cysteine.

These results are fully consistent with what is expected for a protein that has a high homology with two tandem EGF-like motifs (Fig. 1*A*). According to a BLAST search in PDB, the sequence with a higher degree of sequence identity (47%) is that of the Notch ligand Δ-like 1 protein (4xbm) (38) (residues 288–368). In the two tandem EGF domains of PDB 4xbm only five disulfide bridges of the six expected for these motifs were observed (Fig. 1*B*). The sixth bridge between Cys^332^ and Cys^343^ is not formed because the two cysteines are slightly too far (4.5 Å apart S-S distance) but still relatively close. The results from spectrometry mapped onto a model built using PDB 4xbm as a template indicated that most the disulfide bridges of HT-Pvfp-5β match those observed in PDB 4xbm. However, the N-terminal one is not formed possibly because it is to close to the fraying edge of the domain (Fig. 1*C*). Interestingly, the conformation of the linker between the two EGF motifs, which contains Cys^65^, does not allow formation in the model of the sulfur bridge Cys^65^–Cys^76^ as the consequence of a two-residue deletion. Because we observe it experimentally, we must deduce that the accuracy of the homology modeling in this region is low. A more realistic model might be obtained by imposing in the modeling the sulfur bridge as a restraint.

### Probing the fold of recombinant HT-Pvfp-5β

**Figure 2.**
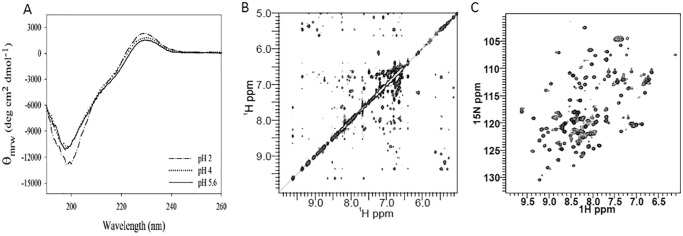
**Structural characterization of HT-Pvfp-5β.** *A,* CD spectrum of the protein in 5% acetic acid (pH 2.0), 0.1 m sodium acetate buffer (pH 4.0), and 0.1 m sodium acetate buffer (pH 5.6). The final concentration of the tested protein was 34 μm. All spectra were corrected by solvent subtraction. Spectra were collected after each new preparation of the protein (at least 12) to check reproducibility. *B,* a portion of the homonuclear 2D NOESY spectrum. *C,* ^15^N HSQC NMR spectrum of HT-Pvfp-5β. Both NMR spectra were recorded in H_2_O/D_2_O (95%/5% in volume) at pH 2.4, at 25 °C, and 600 MHz.

We then investigated the secondary and tertiary structure of HT-Pvfp-5β. Far-UV CD spectra of the protein dissolved in 5% acetic acid (pH 2.0) exhibited a negative peak at 200 nm and a weak positive maximum at 230 nm, the latter probably caused by the high content of tyrosines (17.5%) (Fig. 2*A*). Spectral deconvolution gave predominant content of β-sheet and random coil conformations (Table S1). The effect of pH on the CD spectrum of HT-Pvfp-5β was investigated in the range of 2.0–5.6. The spectra did not change except for a minor increase of the maximum at 230 nm and a small deepening of the minimum at 200 nm at less acidic pH. The spectrum is very similar to that published for HT-Pvfp-5β directly purified from mussels (32), confirming that the recombinant protein has properties similar to those observed for the protein from natural sources. Because, however, the CD spectra are far from what is expected for a predominantly β-sheet protein (39), we considered these results inconclusive for deciding whether the protein has the EGF-like fold as expected. We resorted instead to NMR spectroscopy. We recorded a ^1^H two-dimensional NOESY spectrum of HT-Pvfp-5β, which showed an excellent distribution of resonances with amide protons resonating up to 9.6 ppm and ring current shift effects that are typical of a well-folded protein with a defined hydrophobic core (Fig. 2*B*). The spectrum also revealed several clearly defined connectivities directly left to the water signal (5–6 ppm) as expected for a β-rich protein such as EGF-like domains (40). The general appearance of the spectrum is that expected for a nonaggregated protein. Similar conclusions were supported by a HSQC spectrum (Fig. 2*C*), where it is possible to count 90 peaks of the 95 expected. Apart from some expected changes in the chemical shifts, no other drastic changes in the general dispersion and linewidths were observed in the pH 2.0–7.4 range that could indicate aggregation (data not shown). Thus, our data directly support for the first time the prediction so far solely based on sequence similarity that the Pvfp-5β protein is able to fold as a stable EGF-like domain.

### Surface coating using HT-Pvfp-5β

**Figure 3.**
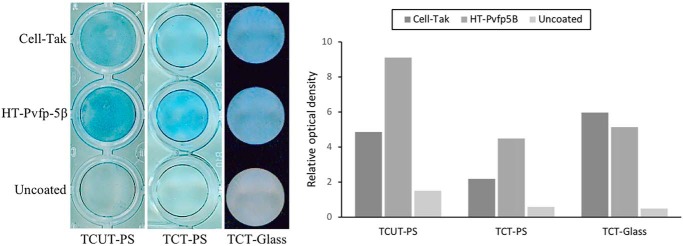
**Coating analysis of HT-Pvfp-5β on polystyrene and glass surfaces.** Cell-Tak and uncoated wells were used as positive and negative controls, respectively. The figure shows the Coomassie Blue staining (*left*) and the relative densitometry analysis (*right*) of the coated proteins. The image is representative of five independent experiments.

We then investigated the surface-coating ability of purified HT-Pvfp-5β. The method is based on the observation that mfps come out of solution as pH is raised and spontaneously adsorb and coat to the first surface they contact. Three surfaces normally used in cell culture were tested: a hydrophilic surface (tissue culture-treated polystyrene (or TCT-PS) 96-well-plate), a hydrophobic surface (untreated polystyrene (or TCUT-PS) 96-well-plate), and a tissue culture-treated glass 96-well-plate (TCT-G). Cell-Tak, a natural extract of the *Mytilus edulis* adhesive-protein mixture of mfp-1 and -2, and uncoated wells were used as positive and negative controls, respectively. Adsorption and coating of HT-Pvfp-5β and Cell-Tak in 5% acetic acid were performed based on Cell-Tak manufacturer's instruction (adsorption method) adding sodium bicarbonate at pH 8.3. HT-Pvfp-5β showed remarkable coating ability for all surfaces (Fig. 3). A staining more pronounced than with Cell-Tak was observed on both polystyrene surfaces.

### pH-induced self-assembly of HT-Pvfp-5β

**Figure 4.**
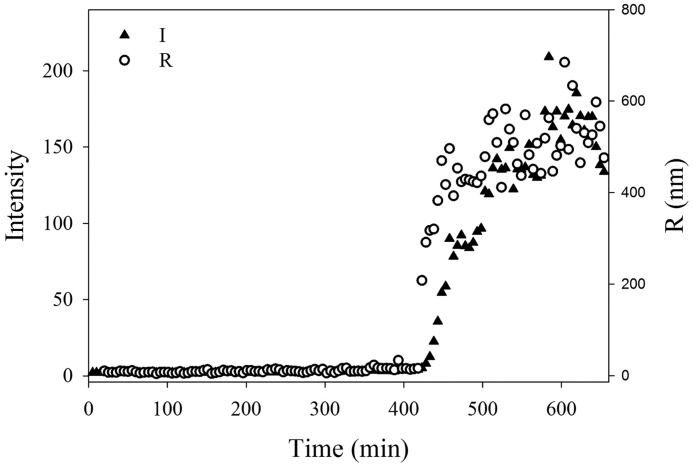
**Time course of light scattered intensity (*I*) and hydrodynamic radius (*R*) for a sample of HT-Pvfp-5β (protein concentration 5 μm) after changing the pH from 2.0 to 6.8 by addition of an appropriate amount of 0.1 m sodium bicarbonate at pH 8.3.** The experiment was repeated on two independent sample preparations.

We reasoned that given the observed coating properties, it would be reasonable to assume that Pvfp-5β has a tendency to self-assemble. On the other hand, the protein is clearly stable in a wide range of concentrations as judged both by CD and NMR. We tried to identify the conditions that could promote aggregation in the coating assay using DLS and followed the behavior of the protein in aqueous solution at each step of the surface-coating protocol. HT-Pvfp-5β (125 μm) proved to be fully soluble in a 5% acetic acid aqueous solution at pH 2. No signal of the autocorrelation function was detectable by DLS measurements as expected for a well-dispersed solution of a protein with a hydrodynamic radius of only a few Å. No signal was detected after dilution, up to 19 μm. Upon addition of an appropriate amount of 0.1 m sodium bicarbonate at pH 8.3, the pH suddenly changed to 6.8 due to generation of CO_2_. Under these conditions of pH shock, small objects of about 10 nm were observed after a few minutes. They remained stable until ∼400 min. After that, a sudden increase of intensity and size was simultaneously observed. The curve profile was typical of a phase transition triggered by a sufficient concentration of critical nuclei formed during the lag phase (supersaturation) (Fig. 4). At the end of the aggregation kinetics, the sample showed a heterogeneous population of low and high molecular weight objects as confirmed by SE imaging (data not shown). These results suggest a propensity of the protein to aggregate under conditions of pH shock.

### Cell proliferation on different HT-Pvfp-5β–coated surfaces

**Figure 5.**
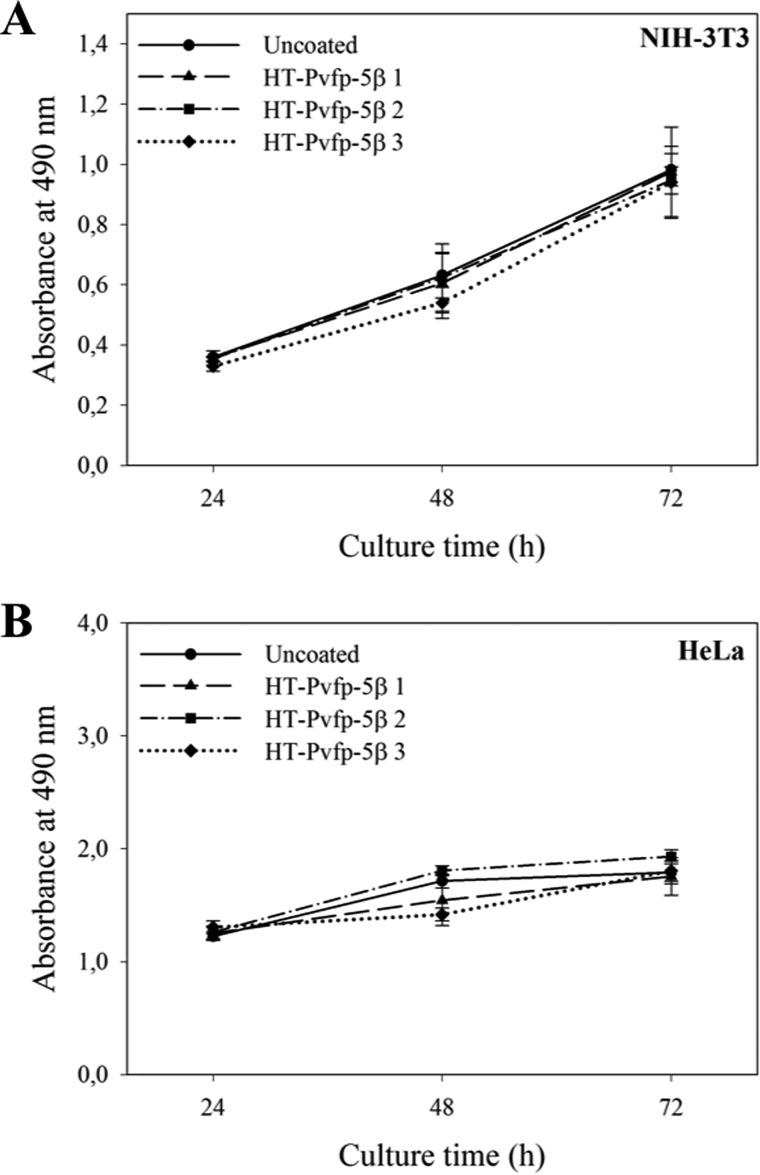
**Cell viability of NIH-3T3 and HeLa cells grown on HT-Pvfp-5β coating using MTS assay.** Three coating concentrations were used for HT-Pvfp-5β (1.75 μg/cm^2^ of Pvfp-5β 1; 3.5 μg/cm^2^ of Pvfp-5β 2; 7 μg/cm^2^ of Pvfp-5β 3). Uncoated surface was used as negative control. NIH-3T3 and HeLa cells in serum-containing medium were seeded at a density of 5 × 10^3^/well and incubated for 72 h; (*A*) NIH-3T3 cells; (*B*) HeLa cells. Each value represents the mean ± S.D. of three independent experiments.

The surface-coating ability of HT-Pvfp-5β led us to investigate the effect of HT-Pvfp-5β coating on cell proliferation and viability. To assess this, we performed the MTS assay, a colorimetric method for sensitive quantification of viable cells in proliferation and cytotoxicity. The method is based on the reduction of the MTS tetrazolium compound by viable cells (41). This reaction generates formation of a colored formazan product in cell culture media that is induced by NAD(P)H-dependent dehydrogenase enzymes. The assay was performed throughout 72 h of cell growth. Absorbance of the formazan dye produced in the cell culture plates was evaluated in triplicates in a microplate reader at 490-nm wavelengths. The obtained absorbance values were directly proportional to the number of viable cells in the culture. Cells were seeded into uncoated or HT-Pvfp-5β–coated wells of TCT-PS 96-well-plates. Both NIH-3T3 and HeLa cells remained viable for 72 h after being in contact with HT-Pvfp-5β. No statistically significant differences were observed between uncoated or coated HT-Pvfp-5β wells, indicating no cytotoxic effects of HT-Pvfp-5β coating (Fig. 5).

### Cell adhesion and spreading of HT-Pvfp-5β

**Figure 6.**
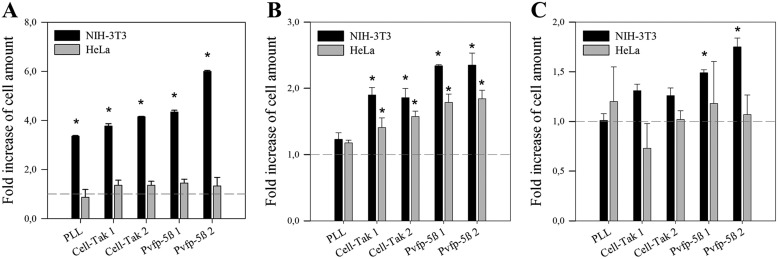
**Cell adhesion of NIH-3T3 and HeLa cells on different surface materials coated with PLL or HT-Pvfp-5β.** *A,* tissue culture-treated glass plate (*TCT-G*); *B,* tissue-culture untreated polystyrene plate (*TCUT-PS*); *C,* tissue-culture treated polystyrene plate (*TCT-PS*). Two coating concentrations were used for HT-Pvfp-5β and Cell-Tak (3.5 μg/cm^2^ of Pvfp-5β 1 and Cell-Tak 1; 7 μg/cm^2^ of Pvfp-5β 2 and Cell-Tak 2), PLL was used as positive control at 7 μg/cm^2^. NIH-3T3 and HeLa cells in serum-free medium were seeded at a density of 5 × 10^4^/well and incubated for 2 h. Fold-increase of the cell amount is referred to the uncoated control for each surface material (*dotted gray line*). The effects were determined by the MTS assay. Each value represents the mean ± S.D. of three independent experiments. Statistical significance: *, *p* < 0.05 *versus* uncoated control.

Finally, we investigated whether the protein promotes cell-adhesion or has spreading abilities. A cell-adhesion assay was performed on different surfaces (glass and polystyrene) with HT-Pvfp-5β coating, Cell-Tak coating, and PLL coating (positive control) and noncoating (negative control). HeLa and NIH-3T3 cells were diluted in serum-free medium to eliminate exogenous cell attachment and spreading factors, and seeded into uncoated and coated wells of TCT-PS, TCUT-PS, and TCT-G 96-well-plates. After 2 h of cell adhesion, the unattached cells were aspirated gently and rinsed with PBS. The MTS assay was performed to measure the living-cell adhesion quantitatively. HT-Pvfp-5β exhibited better cell-adhesion ability than PLL and Cell-Tak on glass and polystyrene plates with both cell lines. The number of adherent NIH-3T3 cells increased by about 4.2–6-fold on the HT-Pvfp-5β–coated glass surface compared with the uncoated glass (Fig. 6*A*) and by 1.5–2.3-fold on HT-Pvfp-5β–coated polystyrene surfaces (Fig. 6, *B* and *C*). Although HeLa cells appeared more spread on all surfaces coated with HT-Pvfp-5β the number of adherent cells was higher only on HT-Pvfp-5β–coated tissue-culture untreated polystyrene surfaces (1.8–1.9-fold) (Fig. 6, *A–C*).

**Figure 7.**
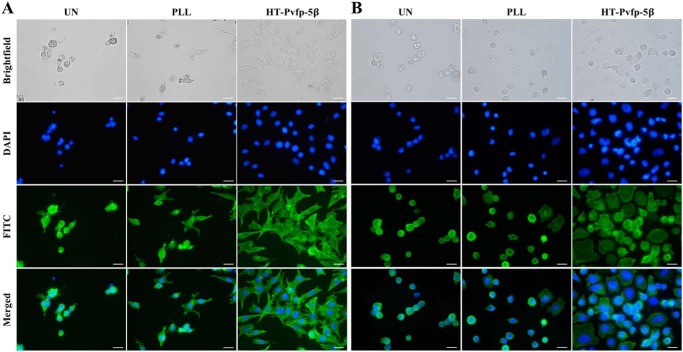
**Cell spreading of NIH-3T3 and HeLa cells on uncoated (*UN*), PLL, or HT-Pvfp5β–coated glass wells.** Fluorescent microscopy images of NIH-3T3 cells (*A*) and HeLa cells (*B*). Cell nuclei was in *blue* (Hoechst staining-DAPI channel) and F-actin was in *green* (Alexa Fluor^TM^ 488-labeled phalloidin staining-FITC channel). *Scale bar*: 20 μm. Three independent experiments were performed.

After cell attachment on the surface, cells underwent morphologic changes and spreading with an active reorganization of the cytoskeleton. This is due to actin polymerization from a monomeric G-actin form to the filamentous polymeric F-actin form that causes rearrangements characterized by formation of protrusion including filopodia and lamellipodia. To study whether the adhesive substrate HT-Pvfp-5β was capable of supporting spreading, F-actin was stained to observe the cytoskeleton. HeLa and NIH-3T3 cells were seeded in serum-free medium, into uncoated PLL or HT-Pvfp-5β–coated glass wells of an 8-well Nunc Lab-Tek II chamber slide. Unattached cells were aspirated and the adhesion state and spreading of attached cells were analyzed by actin staining by the green fluorescent Alexa Fluor^TM^ 488 Phalloidin and fluorescence microscopy. A higher quantity of adherent cells with a more organized cytoskeleton were observed on HT-Pvfp-5β coating in both HeLa and NIH-3T3 cells if compared with the poorly spread cells on PLL-coated wells (Fig. 7 and Table S2). In contrast, on the uncoated surface cells were not spread and F-actin was concentrated along the plasma membrane. These results show that coating of HT-Pvfp-5β is biocompatible and significantly affects cell attachment and spreading.

## Discussion

The development of new biomaterials has a prominent role, which has lately attracted increasing interest for applications in regenerative medicine, biomaterials, and biotechnology. It is not an easy task because, ideally, a polymeric scaffold in biomaterial engineering should have several important features that do not admit a compromise: they should (i) hold appropriate surface properties promoting cell adhesion, proliferation, and differentiation, (ii) be biocompatible and biodegradable, (iii) have high porosity and a high surface-area to volume ratio, with an interconnected pore network for cell growth and flow transport of nutrients and metabolic waste, and (iv) have specific mechanical properties.

Here, we presented a study in which we have characterized Pvfp-5β, one of the main components of the byssus of *P. viridis*. We first set up a scheme for the production of the recombinant protein because this possibility is the prerequisite to produce suitable quantities of the protein in the future without the ordeal of having to purify it directly from mussels. Recombinant production also provides higher flexibility to obtain the protein with and without post-translational modifications and thus the possibility to distinguish the intrinsic properties of the protein from those of the post-translational modifications. The endeavor was not straightforward because Pvfp-5β contains 12 cysteines, which constitutes as much as 15% of its composition. Despite the considerable advancements in the production of cysteine-rich proteins, the task remains challenging. After trying different constructs and optimizing the conditions, we found that a suitable strategy is the expression of the protein in inclusion bodies and its refolding in the presence of GSH.

This meant that we could, for the first time, characterize the protein in its non-DOPA bound form. We found that refolded HT-Pvfp-5β is able to adopt a native conformation with correct pairing of the sulfur bridges according to an EGF-like module as supported by MS and NMR studies. This evidence is new because, whereas our spectrum is in perfect agreement with that of Pvfp-5β directly purified from mussels (32), previous studies were based only on CD data that, for a protein with an EGF-fold, are inconclusive: as observed in model compounds, a CD spectrum with a minimum around 195 nm and a small positive band around 215 nm is the hallmark of a random coil conformation (39). This should be compared with the CD spectrum of a β-protein, which should have a minimum around 215 nm. However, it is well-known that in some small well-folded all-β-sheet proteins such as EGF- and SH3-like domains (42), the CD spectrum is something intermediate between that of a random coil and a β-sheet conformation. This peculiarity is probably the consequence of the combination of internal dynamics, the presence of π-π stacking interactions between aromatics (39), and other similar factors. This is precisely the spectrum we observed for HT-Pvfp-5β.

To prove a β-rich–fold, we resorted instead to NMR. The NMR spectrum of HT-Pvfp-5β has all the features of a folded protein, eliminating any doubt that refolding could have been unsuccessful. The homonuclear 2D NOESY spectrum also supports the presence of a significant β-sheet content as expected for an EGF-like–fold. We are thus confident that the recombinant protein can be used for further studies as a much more ductile substitute of the protein from natural sources.

We characterized the protein for its adhesion and cytotoxicity. Interestingly, we could show that HT-Pvfp-5β retains considerable adhesion properties also in the absence of DOPA. This suggests that these properties are an inherent feature of this mfp rather than solely depending on the presence of DOPA. We also demonstrated that the protein has no tendency to aggregate at acidic conditions, whereas it can readily self-assemble under specific stress conditions. This behavior is generally observed in mfps because of their high levels of aromatic and basic amino acids (43) and is probably a key element to determine the adhesion properties of Pvfp-5β. Finally, we demonstrated that recombinant Pvfp-5β is nontoxic as requested for a biomaterial. Taken together, our study proposes Pvfp-5β as a potential source of biomaterial. Future studies, already on-going in our laboratory, will clarify the biodegradability and the mechanical properties of Pvfp-5β and undertake a detailed comparison of the properties of the native and the post-translationally modified protein.

## Experimental procedures

### Expression and purification of recombinant protein HT-Pvfp-5β

Cloning was achieved as detailed in supporting materials. *E. coli* BL21-Gold(DE3) cells (Agilent Technologies) were grown in Luria-Bertani (LB) medium with 50 μg ml^−1^ of ampicillin (Sigma) and transformed with the vector expressing the recombinant Pvfp-5β as a TEV protease cleavable N-terminal His-tag protein (HT-Pvfp-5β). When *A*_600_ of the culture reached 0.6–0.8, expression was induced by 1 mm isopropyl β-d-thiogalactopyranoside for 3 h at 37 °C and 250 rpm. The harvested cell pellet was washed twice with 100 mm Tris-HCl, 10 mm EDTA, 5 mm CaCl_2_ (pH 7.4) and then with 20 mm sodium phosphate buffer (pH 7.4). The cell pellet was resuspended in pre-chilled lysis buffer (20 mm sodium phosphate buffer, pH 7.4, 500 mm NaCl, 2 mm DTT, MgCl_2_, 5 mm, cOmplete EDTA-free protease inhibitor-Roche mixture, 10 μg/ml of DNase, and 0.5 mg/ml of lysozyme). The cells were disrupted on ice by ultrasonic homogenizers (Bandelin HD 2070), and incubated 30 min at 4 °C. The inclusion bodies containing expressed recombinant HT-Pvfp-5β were washed twice in lysis buffer with 1% Triton X-100, followed by one wash in the same buffer. The washed inclusion bodies were solubilized for 2 h at room temperature then overnight at 4 °C in 8 m urea, 1 m NaCl, 2 mm DTT, 20 mm sodium phosphate buffer (pH 7.4). The supernatant was collected after centrifugation at 20,000 × *g*, 4 °C for 30 min, filtered using 0.45-μm filter (Sartorius Stedim Biotech), and loaded onto a 5-ml HisTrap FF crude column prepacked with Ni-Sepharose (GE Healthcare Life Sciences) equilibrated in the same buffer of the protein sample. The His-tagged protein was eluted under denaturing and reducing conditions with a linear gradient from 0 to 500 mm of imidazole in 10 CV at room temperature. Refolding of the eluted protein was then performed by sequential extensive dialysis at 4 °C, first in 20 mm sodium phosphate (pH 7.4), 2 m urea, 250 mm NaCl, 2 mm reduced gluthatione (GSH), and 0.5 mm oxidized gluthatione (GSSG), then in the same buffer with no urea. Last, refolded HT-Pvfp-5β was dialyzed in 5% acetic acid and lyophilized. Protein purity was verified by 14% (w/v) SDS-PAGE and Coomassie Brilliant Blue staining and resulted in >90%. Protein concentration was assessed by spectrophotometric determination at 280 nm (extinction coefficient (ϵ) = 27570 m^−1^ cm^−1^). The final yield was about 4.3 mg of protein/g of cell pellet for the unlabeled protein and about 1.6 mg of protein/g of cell pellet for the ^15^N-labeled protein.

### Disulfide bridge characterization

The disulfide bridges of refolded HT-Pvfp-5β were identified by the combined use of proteolytic digestion and MS. The freeze-dried protein (10 nmol) was dissolved in 0.1 mm HCl (pH 4), diluted in 100 mm ammonium bicarbonate (final pH 7.5), and incubated with a 100-fold molar excess of iodoacetamide for 30 min in the dark to achieve covalent modification of the cysteine residues not involved in disulfide bonds. The reaction was stopped by removing the unreacted iodoacetamide by SPE cartridge (Chromabond HRX, Macherey-Nagel). An aliquot (0.5 nmol) of the modified HT-Pvfp-5β underwent LC-MS analysis on a Q-TOF premier instrument coupled with an Alliance 695 HPLC pump (Waters) to determinate the number of modified cysteines and verify the homogeneity of the reacted protein. The protein was loaded on an Aeris C4 (150 × 2.1 mm) column (Phenomenex) and eluted by performing a gradient from 20 to 80% of acetonitrile over 5 min. Mass spectra were acquired in positive ion mode over the 1500–3000 *m*/*z* range. The modified protein was then lyophilized, dissolved in 100 mm ammonium bicarbonate (pH 7.5), and incubated with proteomic-grade trypsin (enzyme:protein ratio, 1:50 (w/w)) at 37 °C. After 6 h of reaction, chymotrypsin (enzyme:protein ratio, 1:100 (w/w)) was added to the mixture and the digestion proceeded for a further 2 h and stopped by lowering the pH to 2 with 1 m HCl. The resulting fragments were analyzed by the LC-MS system. Peptide separation was achieved with a Aeris C18 (150 × 2.1 mm) column (Phenomenex) and a gradient from 5 to 65% of acetonitrile over 30 min. Mass spectra were in positive ion mode over the 500–2000 *m*/*z* range; most abundant species underwent MS/MS analyses. Mass and MS/MS spectra were analyzed by the Masslynx software (Waters). The analysis was repeated on 10 independently produced samples to test reproducibility.

### Structural characterization

CD studies were carried out with a J-815 (Jasco, Tokyo, Japan) spectropolarimeter using a quartz cell with 0.1-mm path length. Spectra were recorded at 20 °C with a scan rate of 100 nm/min averaged over five times. The final concentration of the tested protein was 34 μm. All spectra were corrected by subtracting the spectra of the solvent.

NMR spectra were recorded at 25 °C on a Bruker AVANCE III^TM^ HD operating at 600 MHz ^1^H frequency. A NOESY spectrum was recorded on 250 μm sample in H_2_O/D_2_O at pH 2.4 using a mixing time of 150 ms. HSQC spectra measurements were carried out using a ^15^N uniformly labeled protein at a concentration of 250 μm and different pH values (from 2.4 to 7.4). The spectra were processed using the NMRPipe program (36).

DLS measurements were performed using a Brookhaven Instrument BI200-SM goniometer with temperature controlled by a thermostatic recirculating bath. The light scattered intensity and time autocorrelation function were measured at 90° scattering angle by a Brookhaven BI-9000 correlator and a 50 milliwatt He-Ne laser tuned at λ = 632.8 nm. The correlator operated in the multi-τ mode and the experimental duration was set to >2000 counts on the last channel of the correlation function. Data were fit by the cumulant method (37) to derive a z-averaged translational diffusion coefficient, which was converted to an average apparent hydrodynamic radius of an equivalent sphere, *R_h_*, through the stoke-Einstein relationship: *D_z_* = *kT*/6πη*R_h_* (where *k* is the Boltzmann's constant, *T* the absolute temperature, η the viscosity, and *D_z_* the translational diffusion coefficient). Before DLS measurements, all solutions were filtered through 0.2-μm cellulose acetate (Millipore) syringe filters to remove high molecular species. CD, DLS, and NMR measurements were recorded on independent protein preparations to compare the homogeneity of the samples and confirm reproducibility.

### Surface coating

Tissue culture-treated (hydrophilic surface) (Corning) and untreated (hydrophobic surface) (Greiner Bio-One International) polystyrene 96-well-plates (TCT-PS and TCUT-PS respectively), and tissue culture-treated SensoPlate Plus glass bottom 96-well-plates (TCT-G) (Greiner Bio-One International) were coated with HT-Pvfp-5β or Cell-Tak (BD Bioscience), a natural extract of mfps. The amount of coating material used was 7 μg/cm^2^ of well-area. Cell-Tak and uncoated wells were used as positive and negative controls, respectively. Coating with HT-Pvfp-5β and Cell-Tak were performed based on Cell-Tak manufacturer's instructions (adsorption method). The appropriate amount of Cell-Tak or HT-Pvfp-5β dissolved in 5% acetic acid was diluted with Milli-Q water up to 19 μm, 10 μl were placed into each well, and mixed with 3-fold volumes of neutral buffer solution (0.1 m sodium bicarbonate, pH 8.3). After incubation at room temperature for 10–16 h, the solution was aspirated. Wells were washed thoroughly with sterile Milli-Q water for 1 h. The coating of attached proteins was visualized by Coomassie Blue staining. Five independent experiments were performed with two replicates per each sample.

### Mammalian cell culture

Human HeLa (ACC-673-DSMZ) and murine NIH-3T3 (Sigma) cell lines were cultured in Dulbecco**'**s modified Eagle's medium-high glucose supplemented with 10% fetal bovine serum and 10% bovine calf serum, respectively, 1% penicillin and streptomycin (10,000 units/ml and 10,000 μg/ml, respectively) at 37 °C and 5% CO_2_ humidified air. Cells were treated with trypsin (Sigma) at 80% confluence using the standard protocol, and resuspended into new culture flasks. Cells were passaged every third day.

### Cell proliferation, adhesion, and spreading on HT-Pvfp-5β–coated surface

Cell viability and proliferation were examined by CellTiter 96® AQueous One Solution Cell Proliferation Assay-MTS (Promega). NIH-3T3 and HeLa cells were diluted in serum-containing medium, and seeded into uncoated or HT-Pvfp-5β–coated wells of TCT-PS 96-well-plates at a density of 5 × 10^3^/well. After 24, 48, and 72 h, 20 μl of CellTiter 96® AQueous One Solution Cell Proliferation Assay (Promega) were pipetted into each well. Plates were incubated at 37 °C for 3–4 h in a humidified 5% CO_2_ atmosphere. Absorbance was read at 490 nm using a multiwall plate reader (BioRadiMark^TM^ Microplate Reader). The obtained absorbance values were directly proportional to the number of viable cells in culture.

MTS assays were also applied for quantitative cell-binding measurement after cell adhesion on coated substrates. HeLa and NIH-3T3 cells were diluted in serum-free medium, and seeded into uncoated and coated wells of TCT-PS, TCUT-PS, and TCT-G 96-well-plates as described before, at a density of 5 × 10^4^/well. After incubation at 37 °C for 2 h, the unattached cells were aspirated gently with PBS, 200 μl of fresh serum-containing medium were added and the MTS assay was immediately performed as described in the cell proliferation assay. Each MTS assay was performed at least in triplicate.

In cell-spreading assays, HeLa and NIH-3T3 cells were diluted in serum-free medium, seeded at a density of 1.1 × 10^5^/well into uncoated and PLL or HT-Pvfp-5β–coated wells of an 8-well Nunc Lab-Tek II chamber slide. After incubation at 37 °C for 2 h, the unattached cells were aspirated gently with PBS. The adhesion and spreading of cells were analyzed by actin filaments staining by using the green fluorescent Alexa Fluor^TM^ 488 Phalloidin (Invitrogen). Briefly, the adherent cells were fixed with 4% paraformaldehyde for 20 min, permeabilized with 0.5% Triton X-100 in PBS for 10 min, and blocked for 30 min with 5% normal goat serum (Sigma), 0.1% Triton X-100 in PBS. Alexa Fluor^TM^ 488 Phalloidin (1:250) in blocking solution was added and incubated for 1 h in the dark. Then, cells were rinsed twice with PBS and stained with Hoechst 33342 fluorescent DNA-binding dye at 0.01 mg/ml at room temperature for 10 min. After rinsing three times with PBS, the chamber was removed and the slide sealed for the microscopic inspection. The nuclear morphology and cytoskeleton were observed on a Nikon Eclipse 80i microscope equipped for epifluorescence and recorded by a digital camera system.

### Statistical analysis

Values obtained for the cellular analysis were reported as the mean ± S.D. of three independent experiments. Results were analyzed performing one-way analysis of variance with pairwise comparisons using Tukey's HSD test. Analyses were carried out using SigmaPlot 11.0 software (Systat Software Inc., San Jose, CA). Results were considered statistically significant at *p* < 0.05.

### Structure modeling

To obtain a working structural model of Pvfp-5β, a BLAST search (blast.ncbi.nlm.nih.gov/Blast.cgi) was run in the PDB database with the Pvfp-5β sequence. The search identified the region between amino acids 288 and 368 of PDB 4xbm as the closest hit having 47% sequence identify and an *E*-value of 1*e*^−13^. This is the X-ray crystal structure of the Notch ligand Δ-like 1 protein. We then submitted the alignment to the SWISSMODEL server (swissmodel.expasy.org) using our alignment.

## Author contributions

R. S. and A. Pastore conceptualization; R. S., P. L. S. B., D. G., and A. Pastore supervision; R. S., F. V., F. D. P., M. A. M., A. Provenzano, E. R., M. A. C., D. B., P. L. S. B., D. G., A. S., C. A., R. P., and A. Pastore investigation; R. S. and A. Pastore writing-review and editing; A. Provenzano and E. R. data curation; M. A. C., D. B., C. A., and R. P. formal analysis; P. L. S. B. and D. G. resources; A. S. and C. A. methodology; C. A. and R. P. funding acquisition.

## Supplementary Material

Supporting Information

## Footnotes

4 The abbreviations used are:

mfp: mussel foot protein
DOPA: 3,4-dihydroxyphenyl-l-alanine
DLS: dynamic light scattering
EGF: epidermal growth factor
TEV: tobacco etch virus
HSQC: heteronuclear single quantum coherence
PDB: Protein Data Bank
TCT: tissue culture-treated
MTS: 3-(4,5-dimethylthiazol-2-yl)-5-(3-carboxymethoxyphenyl)-2-(4-sulfophenyl)-2*H*-tetrazolium
PLL: poly-l-lysine.
