# Supplementary material for: Recombinant mussel protein Pvfp-5β: A potential tissue bioadhesive
Source: J Biol Chem. 2019 Jul 10;294(34):12826–35. doi: 10.1074/jbc.RA119.009531 (PMC6709630; doi:10.1074/jbc.RA119.009531)
Supplement: Supporting Information [file supp_294_34_12826__index.html]

Recombinant mussel protein Pvfp-5β: a potential tissue bioadhesive — Characterization of the recombinant mussel protein Pvfp-5β — Recombinant mussel protein Pvfp-5β: A potential tissue bioadhesive — Characterization of the recombinant mussel protein Pvfp-5β — Supporting Information 

# Recombinant mussel protein Pvfp-5β: A potential tissue bioadhesive

## Supporting Information

- Supporting Information (to be published online) - Suppl. Materials
