# Supplementary material for: Recombinant mussel protein Pvfp-5β: A potential tissue bioadhesive
Source: J Biol Chem. 2019 Jul 10;294(34):12826–35. doi: 10.1074/jbc.RA119.009531 (PMC6709630; doi:10.1074/jbc.RA119.009531)
Supplement: Supporting Information [file supp_RA119.009531_153120_2_supp_359913_phf78p.pdf]

Supplementary Materials

Strains and vector construction

The 6xHistag-thioredoxin-TEV cleavage site-Pvfp-5β fusion construct (phTt-Pvfp-5β) was obtained by sequence ligation independent cloning (SLIC). The destination plasmid phTt, an in-house derivative of pMAL-c5x (New England Biolabs), encodes a hexahistidine tag followed by thioredoxin and a Tobacco Etch Virus (TEV) protease site under the control of a ptac promoter. The Pvfp-5β dsDNA insert was synthesised by Integrated DNA Technologies. The sequence of Pvfp-5β was optimised for *E. coli* codon usage and 5'- and 3'- homologous sequences were added to achieve insertion downstream to the TEV cleavage site of phTt. The phTt plasmid was amplified by inverse PCR using KAPA-Hifi Hotstart (Kapa Biosystems, Roche), digested with 20 U of DpnI at 37°C for 2 h (New England Biolabs) and purified with Monarch® DNA Cleanup kit (New England Biolabs). Linear phTt and insert were mixed in a 1:4 ratio in NEB2.1 buffer (New England Biolabs) and 0.5 U of T4 DNA polymerase were added. The exonuclease reaction was allowed for 1 min at room temperature, followed by annealing in ice for 15 minutes and transformation in DH5α competent cells. The plasmid pht-Pvfp-5β, encoding a hexa-histidine tag followed by a TEV cleavage site and Pvfp-5β (hereafter referred as HT-Pvfp-5β), was obtained via deletion of the thioredoxin gene of phTt-Pvfp-5β. The template plasmid was amplified using a pair of mutually complementary primers annealing to the DNA regions flanking the thioredoxin coding sequence. The resulting nicked DNA vector was digested with DpnI and transformed into XL10-Gold ultracompetent cells. The nucleotide sequence of all the plasmids was confirmed by Sanger sequencing.

Figure S1. Over-expression and purification analysis of recombinant HT-Pvfp-5β in *E. coli.* Coomassie blue stained SDS-PAGE analysis. (A) Pilot expression of recombinant HT-Pvfp-5β. M, protein marker; WN and WI, whole cell lysates from BL21(DE3) harboring the pht-Pvfp-5β, before and after induction with IPTG, respectively; IS, insoluble cell fraction; S, soluble cell fraction. The missing lanes between M and WN were samples of a pilot expression of a different recombinant protein.

(B) Analysis of immobilized metal affinity purification of recombinant HT-Pvfp-5β. M, protein marker; 1, solubilized inclusion bodies; 2, flow-through fraction; 3, washout fraction; 4, pooled eluted fractions. The missing lanes between lanes 2 and 3 were the first two washout fractions of the immobilized metal affinity purification of the recombinant HT-Pvfp-5β.

(C) Purity of the refolded HT-Pvfp-5β protein and TEV protease digestion. M, protein marker; 1, HT-Pvfp-5β in PB pH 7.4; 2, HT-Pvfp-5β in PB pH 7.4 plus TEV; 3, HT-Pvfp-5β in 0.1 M

sodium acetate buffer pH 5.6 plus TEV; 4, HT-Pvfp-5β in 0.1 M sodium acetate buffer pH 4 plus TEV; 5, HT-Pvfp-5β in 5% acetic acid pH 2.0 plus TEV. The missing lanes between lanes were loaded with HT-Pvfp-5β as in lane 2 but cleaved with half the amount of TEV protease. (D) Primary structure of HT-Pvfp-5β, bold. The predicted EGF-like domains are underlined. The conserved cysteines are indicated by asterisks, the TEV cleavage site is indicated by a box.

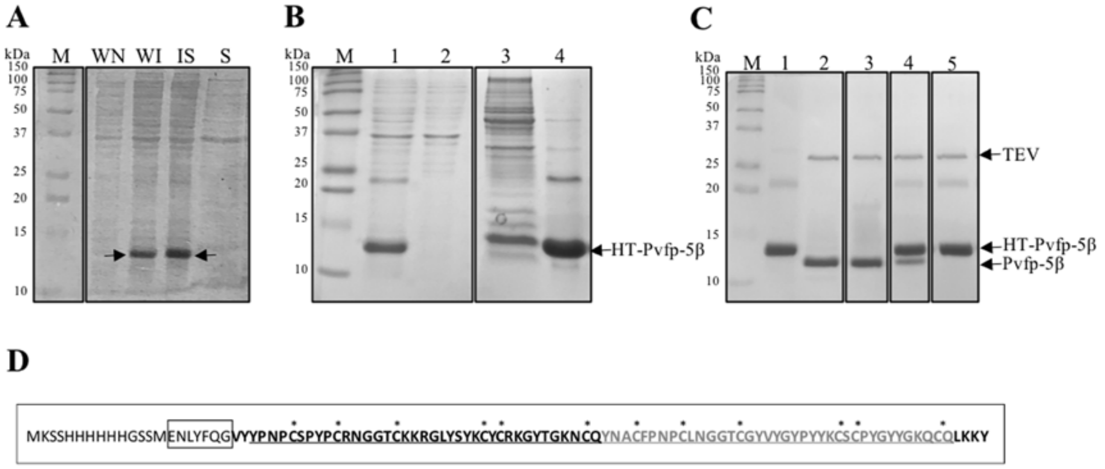

Table S1 – Secondary structure content in percentage of tagged and untagged Pvfp-5β as estimated by the CONTIN software. The differences are well within experimental error.

**Pvfp-5β**	**Helix**	**Strand**	**Turn**	**Unordered**
Tagged	5.3	34.5	22.4	37.8
Untagged	5.3	35.3	22.6	36.8

Table S2 – Quantification of the cell viability experiments shown in Figure 5.

Protein coating	**Cell viability (%)**					
	**NIH-3T3 cells**			**HeLa cells**		
	**24 h**	**48 h**	**72 h**	**24 h**	**48 h**	**72 h**
Uncoated	100.0 ± 0.7	100 ± 12	100 ± 5	100 ± 3	100 ± 4	100 ± 6
Pvfp-5β 1	99 ± 7	96 ± 16	99 ± 15	102 ± 5	90 ± 13	98 ± 9
Pvfp-5β 2	98.3 ± 1.8	99 ± 18	96 ± 4	103 ± 5	105 ± 3	108 ± 3
Pvfp-5β 3	91 ± 4	85 ± 8	96 ± 12	106 ± 5	83 ± 3	101 ± 5
